# Testosterone disrupts human collaboration by increasing egocentric choices

**DOI:** 10.1098/rspb.2011.2523

**Published:** 2012-02-01

**Authors:** Nicholas D. Wright, Bahador Bahrami, Emily Johnson, Gina Di Malta, Geraint Rees, Christopher D. Frith, Raymond J. Dolan

**Affiliations:** 1 Wellcome Trust Centre for Neuroimaging, Institute of Neurology, University College London, 12 Queen Square, London WC1N 3BG, UK; 2 Department of Government, London School of Economics, Houghton Street, London WC2A 2AE, UK; 3 UCL Institute of Cognitive Neuroscience, University College London, Alexandra House, 17 Queen Square, London WC1N 3AR, UK; 4 Interacting Minds Project and Centre of Functionally Integrative Neuroscience, Aarhus University Hospital, Norrebrogade 44, Building 10 G, 8000 Aarhus C, Denmark; 5 Institute of Anthropology, Archaeology and Linguistics, Aarhus University, Aarhus, Denmark

**Keywords:** collaboration, testosterone, information aggregation, social

## Abstract

Collaboration can provide benefits to the individual and the group across a variety of contexts. Even in simple perceptual tasks, the aggregation of individuals' personal information can enable enhanced group decision-making. However, in certain circumstances such collaboration can worsen performance, or even expose an individual to exploitation in economic tasks, and therefore a balance needs to be struck between a collaborative and a more egocentric disposition. Neurohumoral agents such as oxytocin are known to promote collaborative behaviours in economic tasks, but whether there are opponent agents, and whether these might even affect information aggregation without an economic component, is unknown. Here, we show that an androgen hormone, testosterone, acts as such an agent. Testosterone causally disrupted collaborative decision-making in a perceptual decision task, markedly reducing performance benefit individuals accrued from collaboration while leaving individual decision-making ability unaffected. This effect emerged because testosterone engendered more egocentric choices, manifest in an overweighting of one's own relative to others' judgements during joint decision-making. Our findings show that the biological control of social behaviour is dynamically regulated not only by modulators promoting, but also by those diminishing a propensity to collaborate.

## 1. Introduction

Collaborative efforts, for example, when lions hunt in prides or human scientists toil together in the laboratory, can provide benefits to the individual and the wider social group [1–3]. In perceptual decisions, human groups can achieve a performance benefit by combining individuals' information [4], and the potential for benefits from such information aggregation by groups is an important concept in disciplines like political science [5]. Similar benefits from collaboration can accrue to groups in tasks assaying intelligence [6], and collaborative efforts also underlie many cooperative behaviours in choices over the division of resources such as food or money [1,7]. However, a tension exists between collaborative and more self-oriented behaviours: for example, while groups may benefit from a collective intelligence [6] they can be subject to problems such as ‘group-think’ [8]. Previous work on biological factors influencing this balance has identified factors that promote collaboration (e.g. the hormone oxytocin [9] and neural reward mechanisms [10,11]). Instead, here we test whether a candidate agent, the hormone testosterone, can diminish collaboration.

Testosterone is implicated in a variety of social behaviours, and these data point to a potential to diminish collaboration. Higher endogenous testosterone correlates with increased anti-social behaviour in female prisoners [12], higher aggression [13] and more punitive reactions to unfair offers in a bargaining game [14]. Consistent with a potential to disrupt social collaboration, administering exogenous testosterone decreases facial mimicry as measured by facial muscle responses to photographs of emotional faces [15]; decreases the ability to infer emotional states from photographs of eyes [16]; and decreases ratings of trustworthiness in photographs of faces [17]. It has been argued that such findings reflect a more general role for testosterone in increasing a motivation to dominate others (i.e. achieve or maintain social status) [18,19]. Increased status-seeking would in turn predict decreased collaboration in that it entails that individuals, by being more assertive, may be less willing to take account of the opinions of others.

However, when identifying testosterone's effects on social choice, it is important to have a control for testosterone's effects on non-social decision making. In individual choice, endogenous testosterone in men and women has been correlated with psychological variables such as attention [20] and economic variables, such as risk-taking [21]. Administering exogenous testosterone has widespread effects on non-social cognition, for example on working memory [22], spatial memory [23] and reward processing [24]. In particular, testosterone's known associations with reward-related processing [21,24,25] can complicate the interpretation of its effects in traditional economic tasks assaying social choice [26]. These concerns motivate a focus here both on collaborative decision-making without an economic dimension, and also on the need to dissociate testosterone's potential effects on social and individual choice.

**Figure 1.**
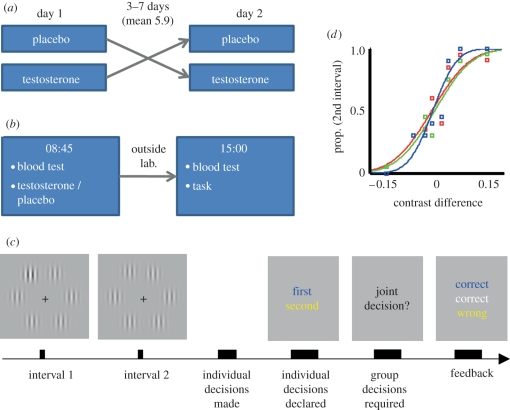
Experimental design. (*a*) Pairs of female participants (dyads) attended on two separate days in a blinded, randomized, placebo-controlled cross-over design. Both dyad members received identical treatment order. (*b*) Participants had blood taken before treatment and testing. (*c*) During testing dyad members sat in the same room viewing separate monitors. In a 2-alternative forced choice, design gratings were presented at two intervals, one containing a target grating with increased contrast. Each participant initially responded without consultation, providing measures of individual decision-making (*S*_indiv_). If they disagreed, a joint decision was requested, which provided a measure of collaborative decision-making (*S*_collective_). (*d*) Example psychometric function for dyad 1 under placebo. Proportion of trials reported as second interval is plotted against target contrast difference. Highly sensitive observers give steep functions with large slope (*S*). Here individuals (*S*_indiv_) are red and green, and the dyad (*S*_collective_) blue.

To isolate the impact of testosterone on collaborative and individual decision-making, we exploited a task that assays each of these components independently [4]. In our task, individuals must share information, and actively collaborate, to gain a performance benefit in a visual perceptual decision task. The task was performed by pairs of participants (dyads) who initially made a perceptual decision alone, enabling us to measure the sensitivity of each individual's non-social decision-making by estimating the slope (*S*_indiv_) of their psychometric function (figure 1). Then, in trials where the dyad's initial responses diverged, one participant announced a collective decision (agreed on via direct verbal negotiation between dyad members), providing a psychometric function for the dyad (*S*_collective_) that reflected collaborative sensitivity. To successfully collaborate, individuals must appropriately weight their own opinion and that of the others prior to a joint decision [5]. We were agnostic about testosterone's potential effects on individual decisions, but predicted that testosterone would causally disrupt collective decision-making.

## 2. Methods

### (a) Participants

Seventeen pairs of participants (dyads) comprised our study sample (mean age 21.7 years, range 18–30; one further dyad was excluded for below-chance behavioural performance). We confined our sample to women, in whom prior evidence links behaviour to both endogenous [12,13,21] and exogenous testosterone [17,26]. All 34 participants were healthy, had normal or corrected to normal visual acuity, took no medication other than long-standing contraceptives (seven took combined oestrogen and progestogen contraception; one took progestogen only contraception), reported regular menstrual cycles (29.1 ± s.d. 2.2 days, range 29–35 days) and were tested between days 1 and 14 of their cycle. All gave informed consent and were paid for attendance.

### (b) Experimental procedure

In a randomized, placebo-controlled, double-blind, cross-over design, 80 mg testosterone undecanoate was administered orally (Restandol testocaps; figure 1*a*). Oral testosterone undecanoate is widely used clinically and has well-known pharmacokinetics [27–29], such that all participants consumed breakfast to aid drug absorption; and the gap between drug administration and the start of behavioural testing was 6–7 h. On two separate days (mean 5.9 days apart, range 3–7 days), the dyad attended at 08.45 when both members received either testosterone or placebo and returned at 15.00 for behavioural testing (figure 1*b*).

Blood samples were taken on each attendance at the laboratory. Total serum testosterone was measured with a standard, commercially available Roche Modular testosterone assay using electrochemiluminescence immunoassay methods in the University College London Hospitals biochemistry laboratory. Biochemical data were available from 14 of the 17 dyads, with hormonal data from the remaining three dyads incomplete owing to administrative errors in the biochemistry laboratory.

### (c) Behavioural methods

In our task, both dyad members sat in a room and performed a 2-alternative forced choice task on identical stimuli presented on separate monitors (figure 1*c* and see the electronic supplementary material for full details). On each trial, there were two intervals and participants initially decided alone in which interval a target (a higher contrast grating) appeared. Target contrast varied between trials, enabling us to measure the sensitivity of each individual's non-social decision-making by estimating the slope (*S*_indiv_) of their psychometric function (figure 1*d*), which was determined using standard methods ([30] and see the electronic supplementary material for details) by plotting the proportion of trials in which the target was reported in the second interval against the contrast difference at the target location (the contrast in the second interval minus the contrast in the first). A large slope indicated highly sensitive performance. After these initial individual decisions, participants then saw their partner's choice. In trials where the dyad's initial responses diverged, one participant was randomly selected to announce a collaborative decision reached after free discussion. As was the case for individuals, we derived a psychometric function for the dyad, where collaborative success was reflected in the slope (*S*_collective_). Feedback either followed the individual decision if they initially agreed, or alternatively followed their joint decision.

### (d) Data analysis

Statistical tests were carried out using paired or independent sample *t*-tests or mixed analyses of variance (ANOVA) in SPSS v. 17.0; reported *p*-values are two-tailed.

## 3. Results

As expected, our hormonal manipulation engendered a large increase in total serum testosterone when comparing the time of behavioural testing (mean 9.3 ± s.d. 9.0 nmol l^–1^) with either morning baseline (1.2 ± s.d. 0.5; paired *t*-test *t*_27_ = 4.7, *p* < 0.0001) or placebo (1.1 ± s.d. 0.6; paired *t*-test *t*_19_ = 4.2, *p* < 0.001). Crucially, testosterone administration had no effect on individual decision-making. Individual sensitivity (*S*_indiv_) under testosterone was no different from placebo when all 34 participants were considered (*S*_indiv_; placebo 3.11 ± s.d. 1.68; testosterone: 2.99 ± s.d. 1.76; paired *t*-test *t*_33_ = 0.5, *p* > 0.6). This was also the case when considering either the better (*S*_max_ placebo 3.80 ± s.d. 1.70; *S*_max_ testosterone 3.69 ± s.d. 1.88; paired *t*-test *t*_16_ = 0.2, *p* > 0.8) or worse performing member of each dyad (*S*_min_ placebo 2.41 ± s.d. 1.38; *S*_min_ testosterone 2.28 ± s.d. 1.33; paired *t*-test *t*_16_ = 0.5, *p* > 0.6). The proportion of trials where the dyad's initial decisions diverged also remained unaffected by testosterone (placebo 0.37 ± s.d. 0.10; testosterone 0.39 ± s.d. 0.08; paired *t*-test *t*_16_ = 0.9, *p* > 0.4).

**Figure 2.**
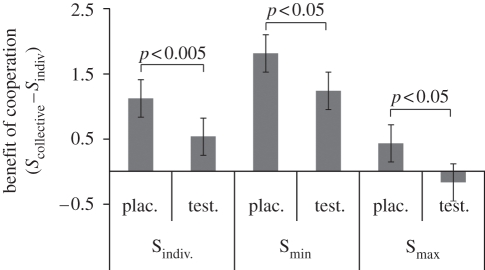
Individuals derive a performance benefit from collaboration. The dyad's collaborative decisions were more sensitive (*S*_collective_) than the individuals' decisions alone (*S*_indiv_). Our metric for this performance benefit on the vertical axis is the difference between an individual's sensitivity and the cooperative sensitivity achieved by their dyad (*Benefit of collaboration* = *S*_collective_ *− S*_indiv_). This benefit is attenuated by testosterone when collapsed across all 34 participants (*S*_indiv_) and also when only the better (*S*_max_) or worse (*S*_min_) members of each dyad are included. All *t*-tests shown are paired. Error bars indicate s.e.m.

Having shown that testosterone did not compromise individual decisions, we could then ask if it had a selective impact on the ability to successfully share information. The logic of effective collaboration is that, if achievable, it benefits the individuals more than acting alone [1–3]. We tested this by asking if testosterone affected the performance benefit each individual accrued from working together, measured by *S*_collective_ *– S*_indiv_ (figure 2). We found that testosterone caused a marked decrease in the individual performance benefit arising from collaboration (*S*_collective_ *– S*_indiv_ placebo 1.13 ± s.d. 1.33, testosterone 0.54 ± s.d. 1.02; paired *t*-test *t*_33_ = 3.3, *p* < 0.005). Furthermore, testosterone disrupted the benefit of collaboration for the better participant (*S*_collective_ *– S*_max_ placebo 0.44 ± s.d. 1.14, testosterone −0.17 ± s.d. 0.59; paired *t*-test *t*_16_ = 2.2, *p* < 0.05) as well as for the worse participant in each dyad (*S*_collective_ *– S*_min_ placebo 1.82 ± s.d. 1.15, testosterone 1.24 ± s.d. 0.86; paired *t*-test *t*_16_ = 2.4, *p* < 0.05). Thus, even from a purely self-interested point of view both dyad members were handicapped when testosterone disrupted the performance benefits from collaboration.

In an evolutionary framework [2,7], our data implicate testosterone as a proximate, mechanistic modulator of collaboration, and specifically one that reduces the ability to collaborate. On this basis, we would expect testosterone to disrupt collaboration via a consistent bias in collaborative decision-making. To test this prediction, we focused on participants' responses as they announced collaborative decisions, where they must appropriately weight each dyad member's opinion. Two considerations might explain how testosterone interferes with this weighting. First, testosterone could lead to a consistent overweighting of the other's opinion, engendering allocentric (other-centred) decision-making, in line with its effect of increasing offers when given in a bargaining game [26]. Second, it could cause consistent overweighting of participants' own opinions, where such egocentricity parallels its effects on trade-offs in animals, for example to eschew parental responsibilities and increase courtship [31,32].

**Figure 3.**
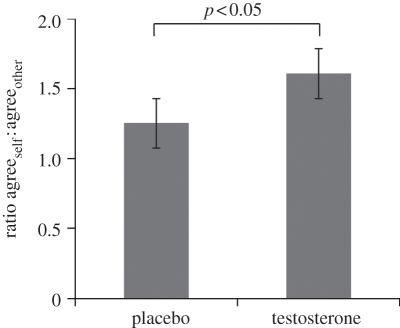
Testosterone disrupts collaboration by increasing the egocentricity of decision-making. Each member of the dyad announced the dyad's joint decision in half the trials where such a collaborative decision was required. The sensitivity of collaborative decision-making hinges on the distribution in weighting attributed to one's own and the other's opinions. For each participant, we measured this weighting by the ratio of times they agreed with themselves (egocentric decisions) to agreement with the other's opinion (allocentric decisions). An egocentric–allocentric ratio of 1 means that participants weight their own and the other's original judgement equally. On placebo, there is trend towards egocentricity bias (one-sample, *t*_33_ = 1.8, *p* < 0.1)—an egocentricity bias that becomes marked on testosterone (one-sample, *t*_33_ = 3.0, *p* = 0.005). We show a paired *t*-test for testosterone versus placebo (*t*_33_ = 2.4, *p* < 0.05). Error bars indicate s.e.m.

To arbitrate between these competing hypotheses, we computed an egocentric–allocentric (E–A) ratio of the number of trials where the announcer agreed with themselves to the number they agreed with the other. Each hypothesis makes a clear prediction: an allocentricity bias decreases the E–A ratio; and an egocentricity bias increases the E–A ratio. Our data fitted predictions from the second hypothesis, namely that testosterone consistently causes an egocentricity bias (figure 3). The E–A ratio increased under testosterone (1.61 ± s.d. 1.17) relative to placebo (1.26 ± s.d. 0.83; paired *t*-test *t*_33_ = 2.4, *p* < 0.05). This increased E–A ratio was consistent across both the best and worst-performing dyad members, as shown in a 2 decision-maker (*S*_min_*, S*_max_) by 2 drug (placebo, testosterone) mixed ANOVA in which there was a main effect of drug (*F*_1,16_ = 5.8, *p* < 0.05) but not decision maker (*F*_1,16_ = 0.1, *p* > 0.7) and no interaction (*F*_1,16_ = 0.6, *p* > 0.4). We also note that this egocentricity bias was not accompanied by altered deliberation time for collective decisions (placebo 7.56 s ± s.d. 3.25; testosterone 7.44 ± s.d. 2.89; paired *t*-test *t*_33_ = 0.5, *p* > 0.6); which in the light of the broader choice literature suggests that the effect was not related to decision uncertainty that is usually accompanied by reaction time changes [33]. Neither E–A ratio nor sensitivity measures were related to total serum testosterone levels (details in the electronic supplementary material). Finally, given a recent study suggesting participants' beliefs about which drug had been administered might affect choice [26]; we tested for this and found no difference in E–A ratio when participants believed they had received placebo (mean = 1.58 ± s.d. 1.18, *n* = 44) compared with when they believed they had received testosterone (1.21 ± s.d. 0.62, *n* = 20; independent samples *t*-test *t*_62_ = 1.3, *p* > 0.1; details in electronic supplementary material).

## 4. Discussion

In our paradigm, testosterone causally and selectively disrupted individuals' ability to successfully collaborate and aggregate their information in order to achieve a performance benefit. Further, this effect was selective because while disrupting collective decision-making, testosterone left individual decisions unaffected, which is important in the light of testosterone's widespread associations with aspects of non-social choice such as attention [20], working memory [22], spatial memory [23] and reward processing [24]. Finally, we demonstrated that, across both the better and worst-performing members of the dyads, testosterone disrupted collaboration by increasing the egocentricity in individuals' choices, operationalized as an enhanced weighting of one's own relative to another's evidence.

Our finding that testosterone increased egocentric choices accords with a broader literature concerning testosterone's role in social choice, and in particular with an interpretation of that literature which proposes that testosterone's role is to increase dominance or status-related behaviours [18,19]. High social status is associated with elevated testosterone in humans [13,19], chimpanzees [34] and other mammals [35]. A greater drive for social status leading to greater assertiveness during social interactions might reasonably be expected to impair an individuals' ability to appropriately weight the opinion of another, consistent with our findings. Indeed, the increased egocentricity in an individual's choices that we observe could be interpreted as a form of signalling, whereby the individual is signalling their dominance in the context of a collective decision.

Increased dominance can be detrimental to collaborative decision-making, as shown previously during reasoning tasks where high variance in the verbal contributions of group members (i.e. groups with highly dominant individuals) led to a significantly attenuated performance benefit from collaboration [6]. Other possible effects of testosterone previously related to its role in status-related behaviour [18] may also contribute to less effective information aggregation in our dyads, for example in reducing trustworthiness ratings of faces [17] and decreasing the ability to infer emotional states through photographs of eyes [16]. In addition to potential status-related effects of testosterone, our finding of increased egocentricity has interesting parallels with testosterone's role in sexual and reproductive behaviours, where testosterone relates to more self-orientated behaviour as evident in reduced parenting and increased courtship in birds [31,32], rodents [36] and rural Senegalese men [37]. Importantly, our task involves no conflict over resources as accurate integration of information is in the best interest of the dyad members, which suggests that the effects of testosterone we observed are not caused by it rendering individuals more selfish.

While the idea that testosterone increases status-related or self-orientated behaviours accords well with the wider literature, future work could usefully examine potential causes of this increased egocentricity in choice that are not addressed in our current study. The observation that testosterone did not affect individual choices militates against explanations for more egocentric choices in terms of general motivational [38] or attentional [20] effects. Because we did not use monetary rewards, this militates against potential explanations in terms of testosterone's known effects on reward processing, which can explain results in more traditional economic paradigms [26]. However, another potential cause of increased egocentricity in individuals' choices is increased confidence in an individual's own original choices, an idea now testable within a framework that assays meta-cognition [39]. A second possibility is that testosterone disrupts collaboration by reducing an individual's ability to signal their confidence, and future work could extend our design such that only one dyad member received testosterone on each day to ask whether one or both dyad members exhibit a bias. A third possibility is that testosterone might render individuals less susceptible to social influence more generally, a potential cause of more egocentric choices that could be explored in variants of classic experiments such as those described by Asch [40].

Social animals reap benefits from collaboration across a wide variety of tasks, ranging from those involving information aggregation (as seen here), reasoning [6] or the division of resources such as food or money [1–3]. Indeed, the potential benefits from information aggregation, for example, are used to support the use of juries (i.e. groups of observers) in the criminal justice system [5]. However, collaborating too freely is not always beneficial, and therefore the biological mechanisms controlling the balance between more collaborative and self-oriented behaviours must dynamically tune behaviour to the social environment. While a previous focus has been on factors promoting collaboration [9–11], here we highlight an opposing biological influence that increases self-orientated or status-related behaviours at the expense of collaboration. Our data show that the humoral agent testosterone modulates the delicate trade-off between collaboration and a more egocentric disposition.
